# Weak Magnetic Internal Signal Characteristics of Pipe Welds under Internal Pressure

**DOI:** 10.3390/s23031147

**Published:** 2023-01-19

**Authors:** Bin Liu, Yanduo Fu, Luyao He, Hao Geng, Lijian Yang

**Affiliations:** School of Information Science and Engineering, Shenyang University of Technology, Shenyang 110870, China

**Keywords:** pipeline weak magnetic stress, simulation analysis, energy factor, experimental test

## Abstract

Weak magnetic detection technology is an effective method to identify stress-induced damage to ferromagnetic materials, and it especially possesses great application potential in long-distance oil and gas pipeline weld crack detection. In the process of pipeline operation, due to internal pressure and external loads, local stress concentration may be generated, and partial stress concentration may lead to local cracks and expansion of the pipe. In order to improve the accuracy of magnetic signal analysis for ferromagnetic materials under internal pressure, the causes of magnetic signal generation at pipeline welds were analyzed from a microscopic perspective. The distributions of magnetic signals at pipeline welds, weld cracks, and base metal cracks under different internal pressures were numerically analyzed. The variation trends of magnetic signal characteristics, such as peak values of axial and radial components, gradient *K*, maximum gradient *Kmax*, and gradient energy factor *S*(*K*), were analyzed. In addition, experiments were carried out to verify the numerical data. It was revealed that with the elevation of internal pressure, the peak values of the axial and radial components, gradient *K*, maximum gradient *Kmax*, and gradient energy factor *S*(*K*) linearly increased. However, the magnitude and average change of *S*(*K*) were larger, which can more directly indicate variations of magnetic signals. The radial growth rate *ν_y_* of *S*(*K*) was 3.24% higher than the axial growth rate *ν_x_*, demonstrating that the radial component of the magnetic signal was more sensitive to variations of stress. This study provided a theoretical and experimental basis for detection of stress-induced damage to long-distance oil and gas pipelines.

## 1. Introduction

Pipelines are the most economic and efficient means of oil and natural gas transportation over long distances in different environments, owing to their advantages of continuous transportation, low cost, high efficiency, climate resilience, and high reliability. Oil and gas pipeline leakages may not only cause serious air, water, and soil pollution, but also result in huge economic losses. According to the pipeline incident statistics published in recent years, weld cracks are one of the main causes of oil and natural gas transportation failure over long distances [1,2], and it is urgent to realize the online detection of small weld cracks (where the opening width is less than 1 mm). At present, in situ pipeline detection technology is the most effective method for testing pipeline safety that is globally recognized by the pipeline industries [3,4,5,6]. Under the normal operation of the pipeline, the internal detection equipment is driven by oil and natural gas (speed, 1–5 m/s) in order to realize the noncontact and dynamic detection of corrosion, cracks, metal loss, pinholes, stress, and other defects in pipelines.

Traditional nondestructive detection techniques, such as eddy currents, magnetic powder, ultrasound, radiation, etc., have shown some deficiencies in pipeline assessment [7,8,9,10]. The internal detection of magnetic flux leakage has become the mainstream technology in the field of pipeline internal detection due to its advantages of being contactless, anti-interference, fast signal acquisition, etc., and the technology can identify a certain opening width of crack defects (where the opening width is greater than 1 mm). However, it is infeasible to effectively and accurately identify microcracks in welded pipes [11,12,13,14,15]. Therefore, research on the internal inspection of microcracks in long-distance welded oil and gas pipelines has noticeably attracted scholars’ attention.

The weak magnetic detection method can realize contactless and dynamic online detection of the stressed areas of ferromagnetic components using the magnetomechanical effect of ferromagnetic materials. It has great application prospects in internal stress detection of pipelines [16]. This technology has been widely used to detect pipeline defects [17,18,19,20,21]. Under internal pressure and external loading of oil and gas pipelines, abnormal stress distribution may occur at the microcracks of welded pipelines. Stress distribution is closely associated with crack size. Using the weak magnetic detection method is advantageous to identify abnormal changes of stress and microcracks in welded pipelines [22,23].

In this study, the causes of magnetic signals generated at welded pipelines were first analyzed from a microscopic perspective. Second, a magnetomechanical model of the composite stressed area was established based on the finite element method, the magnetomechanical effects at the microcracks of a welded pipeline were calculated quantitatively, and the characteristics of weak magnetic signals in pipeline welds, weld cracks, and base metal cracks were studied. In addition, the variation patterns of characteristic parameters of weak magnetic signals, such as gradient value *K*, maximum gradient *Kmax*, and gradient energy factor *S*(*K*) in different stressed areas were analyzed and verified experimentally. This research facilitated detection of microcracks in long-distance oil and gas pipelines.

## 2. Mechanism of Weak Magnetic Internal Detection in Long-Distance Oil and Gas Pipelines

Based on the force magnetic coupling model and the idea of calculus and integration, the spatial distribution model of magnetic signals in pipeline welds, weld cracks, and base metal cracks was established.

### 2.1. Magnetic Detection Mechanism

Based on micromagnetism theory and Weis molecular field theory, the force-magnetic coupling model was derived from the ferromagnetism theory proposed by Jiles and Atherton (J–A theory).

It is assumed that the angle between the magnetic moment of the atom *μ_J_* and the external magnetic field *H* is *θ_i_*, according to the statistics, and the partition function *Z*(*H*) of the system is formulated as follows [24]:

(1) $$Z(H)={\left[\int_{0}^{2\pi }d\Phi \int_{0}^{\pi }e^{\mu_{JH\cos j/KBT}}\sin\theta_{i}d\theta \right]}^{N}={\left[\frac{4\pi K_{B}T}{\mu_{J}H}sh\left(\frac{\mu_{J}H}{K_{B}T}\right)\right]}^{N},$$

where *K_B_* is the Boltzmann constant, *T* denotes the temperature, *N* represents the number of atoms per unit volume, and *θ_i_* is the angle in the range of *0~π*.

According to:

(2) $$\varphi =-K_{B}T\ln Z,$$

and

(3) $$\frac{\partial \varphi }{\partial H}=-M,$$

the magnetization strength *M* can be expressed as:

(4) $$M=K_{B}T\frac{\partial }{\partial H}\left[\ln Z(H)\right]=N\mu_{J}\left[cth\left(\frac{\mu_{J}H}{K_{B}T}-\frac{K_{B}T}{\mu_{J}H}\right)\right],$$

According to:

(5) $$\alpha =\frac{\mu_{J}H}{K_{B}T},$$

*L*(*α*) can be expressed as:

(6) $$L(\alpha )=\mathrm{cth}\alpha -\frac{1}{\alpha },$$

where *L*(*α*) is the Langevin function.

According to the *J–A* theory, a modified Langevin function can be used to fit the magnetization curve of the material:

(7) $$M_{an}=Ms\left[\coth\left(\frac{H+\alpha M_{an}}{a}\right)-\frac{a}{H+\alpha M_{an}}\right],$$

The stress action is equivalent to an additional magnetic field and combines the approach principle to form the stress magnetization model.

The effective field He is expressed as:

(8) $$H_{e}=H+\alpha M+H_{\sigma }=H+\alpha M+\frac{3\sigma }{2\mu_{0}}\left(\frac{d\lambda }{dM}\right),$$

The relationship between the hysteresis expansion coefficient and the magnetization strength of the material can be formulated as follows:

(9) $$\lambda =\left(\gamma_{1}(0)+{\gamma_{1}}^{\prime }(0)\sigma \right)M^{2}+\left(\gamma_{2}(0)+{\gamma_{2}}^{\prime }(0)\sigma \right)M^{4},$$

Then, Equation (8) is simplified as:

(10) $$H_{e}=H+\alpha M+\frac{3\sigma }{2\mu_{0}}\left(\left(2\gamma_{1}(0)+2{\gamma_{1}}^{\prime }(0)\sigma \right)M+\left(4\gamma_{2}(0)+4{\gamma_{2}}^{\prime }(0)\sigma \right)M^{3}\right),$$

It is supposed that the irreversible magnetization follows the law of proximity:

(11) $$\frac{dM_{irr}}{dW}=\frac{1}{\xi }(M_{an}-M_{irr}),$$

where *ξ* is a constant dependent on the energy per unit volume, and *M_irr_* denotes the irreversible component of the magnetization. The derivative of magnetization strength to stress is expressed as:

(12) $$\frac{dM}{d\sigma }=\frac{1}{{}^{\varepsilon^{2}}}\sigma (1-c)(M_{an}--M_{irr})+c\frac{dM_{an}}{d\sigma },$$

Taking Equation (11) into Equation (12), the relationship between stress *σ* and magnetization *M* is obtained as follows:

(13) $$\frac{dM}{d\sigma }=\frac{\frac{\sigma }{E\xi }\left(M_{an}-M\right)+cM_{s}\left[{\mathrm{csch}}^{2}\left(\frac{H_{e}}{a}\right)-\frac{a}{H_{e}^{2}}\right]\left\{\frac{3}{\mu_{0}}\left[\left(\gamma_{1}+\gamma_{1}^{1}\sigma \right)M+2\left(\gamma_{2}+\gamma_{2}^{1}\sigma \right)M^{3}\right]\right\}}{1-cM_{s}\left[{\mathrm{csch}}^{2}\left(\frac{H_{e}}{a}\right)-\frac{a}{H_{e}^{2}}\right]\left[\frac{3\sigma }{\mu_{0}}\left(\gamma_{1}+6\gamma_{2}M^{2}\right)+\alpha \right]},$$

where *α* is the coupling parameter, *H* is the external magnetic field, *σ* denotes stress, *λ* is the magnetostriction coefficient, *M* represents magnetization, and *c* is a reversible coefficient.

**Figure 1:**
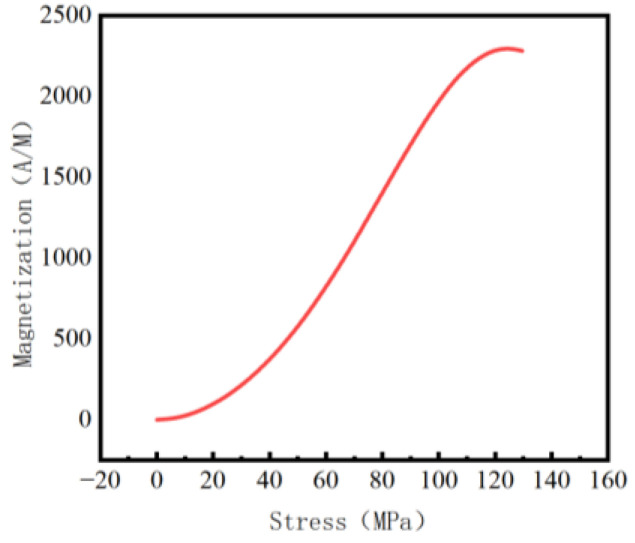
Stress-magnetization curve.

According to Equation (13), material parameters were taken as *c* = 0.25, *µ*_0_ = 4π × 10^−7^ NA^−2^, $\gamma_{1}$ = 7 × 10^−18^ A^−2^∙m^2^, $\gamma_{1}^{1}$ = −1 × 10^−25^ A^−2^∙m^2^∙Pa^−1^, $\gamma_{2}$ = −3.3 × 10^−30^ A^−4^∙m^4^, and $\gamma_{2}^{1}$ = 2.1 × 10^−38^ A^−4^∙m^4^∙Pa^−1^ [25]. The magnetic curve output was plotted as shown in Figure 1. It was revealed that stress corresponded to magnetization one-to-one, and that magnetization increased with the increase of stress (Figure 1).

According to magnetization and the relative permeability *μ_r_:*


(14) $$M=(\mu_{r}-1)\cdot H,$$

Taking Equation (14) into Equation (13), the relationship between relative permeability *μ_r_* and stress *σ* is formulated as follows [26]:

(15) $$\frac{d\mu_{r}}{d\sigma }=\frac{\frac{\sigma }{E\xi }\left[M_{an}-\left(\mu_{r}-1\right)\cdot H\right]+cM_{s}\left[csch^{2}\left(\frac{H_{e}}{a}\right)-\frac{a}{H_{e}^{2}}\right]\cdot \frac{3}{\mu_{0}}\left[\left(\gamma_{1}+\gamma_{1}^{1}\sigma \right)\cdot \left(\mu_{r}-1\right)\cdot H+2\left(\gamma_{2}+2\gamma_{2}^{1}\sigma \right){\left(\mu_{r}-1\right)}^{3}\cdot H^{3}\right]}{\left\{1+cM_{s}\left[csch^{2}\left(\frac{H_{e}}{a}\right)-\frac{a}{H_{e}^{2}}\right]\cdot \left[\frac{3\sigma }{\mu_{0}}\left(\gamma_{1}+6\gamma_{2}{\left(\mu_{r}-1\right)}^{2}\cdot H^{2}\right)+\alpha \right]\right\}H},$$

According to Equation (15), magnetic permeability, as an intermediate value, could effectively establish the coupling relationship between weak magnetic signals and stress, thus directly reflecting the influences of stress on ferromagnetic materials and providing a numerical basis for the following magnetic finite element analysis.

### 2.2. Research on the Weak Magnetic Internal Detection Mechanism of the Pipeline

Weak magnetic internal detection was applied for nondestructive testing of ferromagnetic pipelines using the natural magnetization of the ferromagnetic field. The pipe weld is different from the pipe base metal in metallophase, organization, and stress, thus, the magnetic distribution was obviously different.

The ferromagnetism of the material was linearly correlated with the martensite content [27].

(16) $$M_{sa}=99fM-2.9,$$

where *M_sa_* is the saturation magnetization (emu/g) and *f_M_* is the martensitic volume fraction (%).

In the process of pipe welding, the weld metal goes through three stages: heating, melting, and crystallization and solid phase transformation from the beginning of formation to cooling to room temperature. During welding, the microstructure of the weld material changes, and a large amount of martensite is generated. When the pipeline is running, the loading also induces martensite transformation, and the greater the stress, the more martensite transformation occurs [28,29,30].

**Figure 2:**
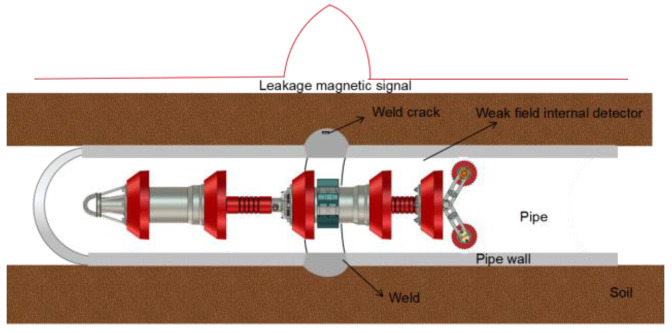
Schematic diagram of in-pipeline detection.

According to Equation (16), the pipe is therefore ferromagnetic in the weld area, and the weak magnetic internal detection technique can identify magnetic signals. The macroscopic manifestation is the sudden change of the self-leakage magnetic field around the weld or weld crack, as shown in Figure 2.

### 2.3. Characteristic Analysis of the Weak Magnetic Signal

Using ANSYS finite element simulation software, the mechanical analysis model of the weld was established in Cartesian coordinate system (x, y, z). Solid70 was used to solve the temperature field. When the grid was divided, the mesh at the weld of the pipeline was finer, 2 mm, and the mesh at both sides of the matrix was thicker, 5 mm. The initial room temperature was 25 °C, and the welding temperature was 1500 °C. The pipe material used in this study was X70, the pipe length was 1000 mm, the outer diameter was 1219 mm, the thickness was 16 mm, the weld width was 20 mm, the welding line speed *V* was 1 mm/s, the welding voltage *U* was 36 V, the welding current *I* was 32 A, and the welding thermal efficiency *η* was 0.75. The expression of heat generation intensity *Q* in unit time was formulated as follows, and *Q* can be calculated by taking corresponding parameters into account [31]:

(17) $$Q=\frac{I\times U\times \eta }{V}$$

The result of temperature field simulation can be read into the stress field simulation to get the result of the stress distribution of the weld.

The von Mises stress *σ_v_* can be expressed as [32]:

(18) $$\sigma_{v}=\sqrt{\frac{1}{2}\left[{\left(\sigma_{\theta }-\sigma_{z}\right)}^{2}+{\left(\sigma_{z}-\sigma_{r}\right)}^{2}+{\left(\sigma_{r}-\sigma_{\theta }\right)}^{2}\right]}$$

where *σ_z_* is axial stress (MPa), *σ_θ_* denotes circumferential stress (MPa), and *σ_r_* is radial stress (MPa).

#### 2.3.1. Weak Magnetic Signals in the Weld Pipe

**Figure 3:**
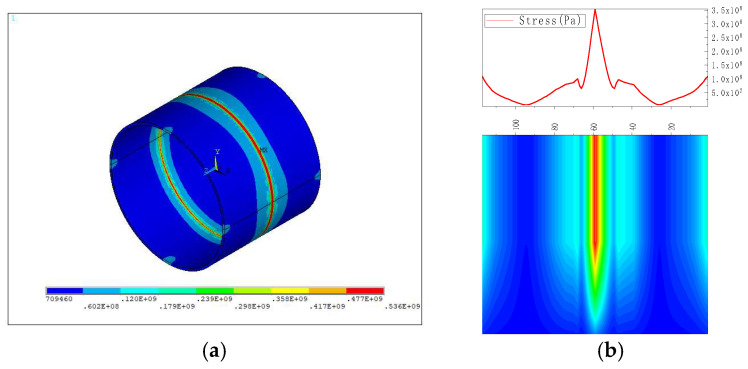
Results of welding stress simulation. (**a**) Welding stress diagram; (**b**) von Mises stress distribution diagram on the extracted y = 0 path.

The welding stress diagram is shown in Figure 3a, and the von Mises stress distribution diagram on the extracted y = 0 path is illustrated in Figure 3b. It was revealed that the stress distribution at the pipe weld was uneven, and there was stress concentration that gradually weakened both sides. The maximum stress was tensile stress, and it was located near the weld, which could justify why the center of the weld would be prone to cracks and other defects.

**Figure 4:**
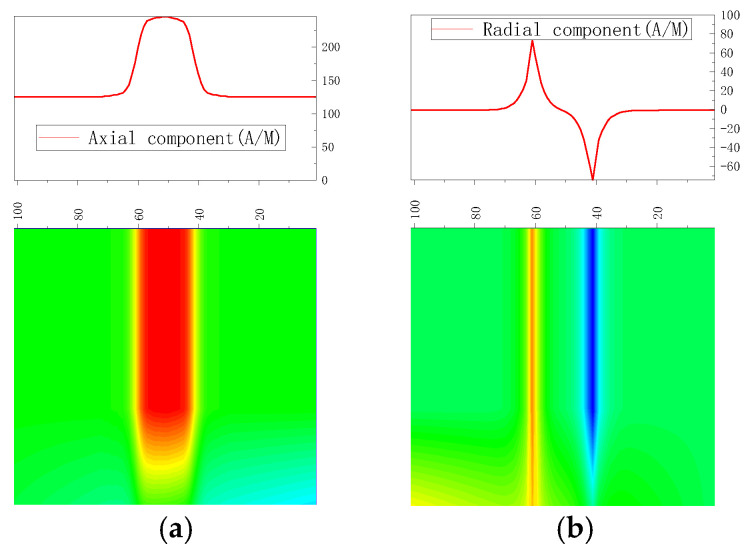
Magnetic signals of the pipe weld. (**a**) Axial component of weak magnetic signal; (**b**) radial component of weak magnetic signal.

As found with Equation (15), the magnetic signal distribution results are shown in Figure 4.

There was a peak in the axial component of the weak magnetic signal at the pipe weld and a sinusoidal fluctuation in the radial component, which is the typical feature of stress concentration, thus confirming the feasibility of detecting stress in the pipe weld using the weak magnetic inner detection method.

#### 2.3.2. Weak Magnetic Signals in Weld Cracks

**Figure 5:**
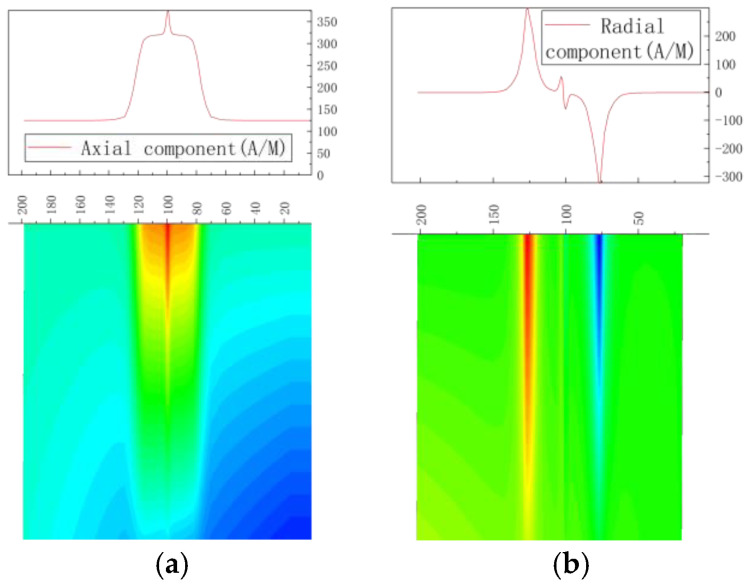
Magnetic signal of weld cracks in the pipe. (**a**) Axial component of weak magnetic signal; (**b**) radial component of weak magnetic signal.

According to the above-described weld simulation model, cracks were created with a size of 2 mm × 0.95 mm × 1 mm (length × width × depth), and the magnetic simulation results are shown in Figure 5.

There was a peak for the axial component of the weld crack and two sinusoidal fluctuations in the radial component. The magnetic signal’s characteristics were compared with the weld magnetic signal (Figure 4), and the weld crack could be identified.

#### 2.3.3. Weak Magnetic Signal of Base Material Crack

**Figure 6:**
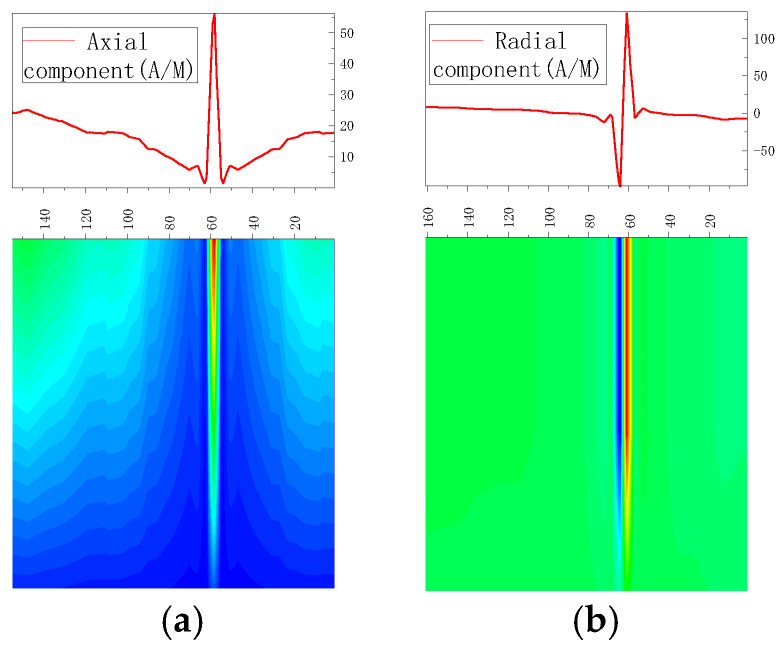
Crack magnetic signal of pipe base material. (**a**) Axial component of weak magnetic signal; (**b**) radial component of weak magnetic signal.

A crack was created with the size of 2 mm × 0.95 mm × 1 mm (length × width × depth) on the pipe base material (consistent with the weld base material parameters). The results of magnetic simulation are shown in Figure 6.

It was revealed that there was a peak in the axial component of weak magnetic signal at the crack of the pipe base material and a sinusoidal fluctuation in the radial component, which is the typical feature of stress concentration, thus confirming the feasibility of detecting cracks using the weak magnetic inner detection method.

## 3. Analysis of Influences of Internal Pressure on Weak Magnetic Signal Characteristics

### 3.1. Influences of Internal Pressure on Weak Magnetic Signal Characteristics of Weld

**Figure 7:**
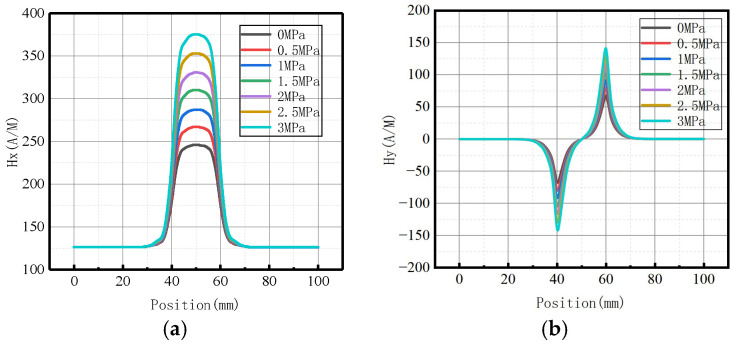
Magnetic signal of weld under different internal pressure. (**a**) Axial component of weak magnetic signal; (**b**) radial component of weak magnetic signal.

According to the pipe weld model established in Section 2.3.1, an internal pressure of 0–3 MPa was applied to the pipe, and simulation was conducted with an interval pressure of 0.5 MPa. The results of testing the weak magnetic signal at the weld are shown in Figure 7.

According to the above-mentioned results of magnetic simulation, the peak values of the axial components and the peak-to-peak values of the radial components, gradient *K*, maximum gradient *Kmax*, and gradient energy factor *S*(*K*) of magnetic signal characteristic parameters were analyzed.

#### 3.1.1. Peak Values in Axial and Radial Components at Weld Varies

**Figure 8:**
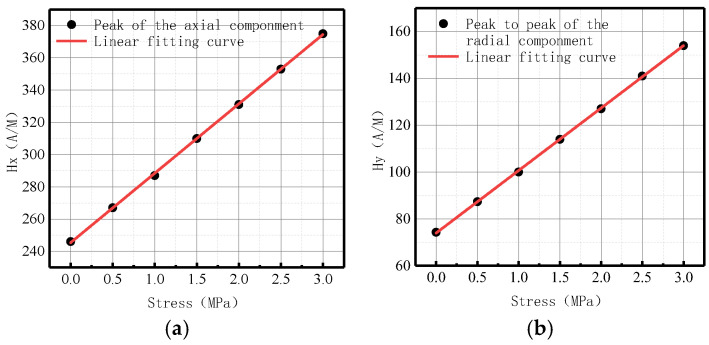
Peak values of weak magnetic signal at weld varies with internal pressures. (**a**) Peak values of the axial component; (**b**) peak-to-peak values of the radial component.

As there were peak values in axial and radial components, the peak values of the axial components and the peak-to-peak values of the radial components changed with the internal pressure as follows (Figure 8):

It was revealed that peak values in axial and radial components increased linearly with the increase of internal pressure. For an increase of pressure with an interval pressure of 0.5 MPa, the average change of peak values in the axial component was 20 A/M, and the average change of peak-to-peak values in the radial component was 13.33 A/M.

#### 3.1.2. Magnetic Field Strength Gradient K at Weld Varies

Due to the stress concentration at the weld, the magnetic field strength at the weld significantly changed, and, accordingly, the magnetic field strength gradient remarkably varied. The magnetic field intensity gradient was calculated as follows:

(19) $$K=\frac{\left|\Delta Hp\left(y\right)\right|}{\Delta lk}$$

where *K* is the gradient value of the magnetic field strength, |Δ*H_p_*(*y*)| is the difference in the magnetic field intensity between the two adjacent detection points, and Δ*l_k_* is the space between two adjacent detection points.

**Figure 9:**
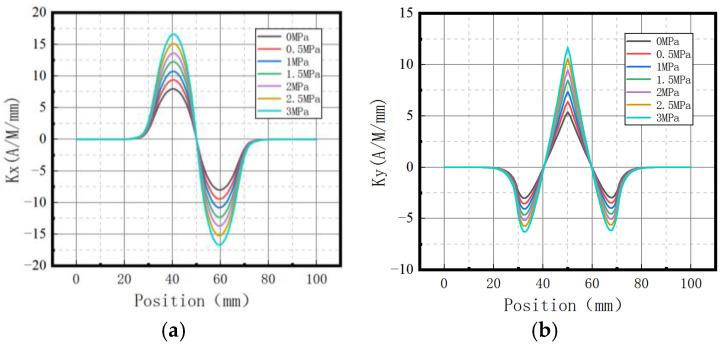
Magnetic signal gradient K variation with internal pressure. (**a**) The axial gradient component *Kx*; (**b**) the radial gradient component *Ky*.

The variations of magnetic signal gradient *K* with internal pressure in the axial and radial components are shown in Figure 9.

It was revealed that the axial gradient component *Kx* and the radial gradient component *Ky* gradually increased with the increase of internal pressure.

#### 3.1.3. Maximum Magnetic Field Strength Gradient Kmax at Weld Varies

**Figure 10:**
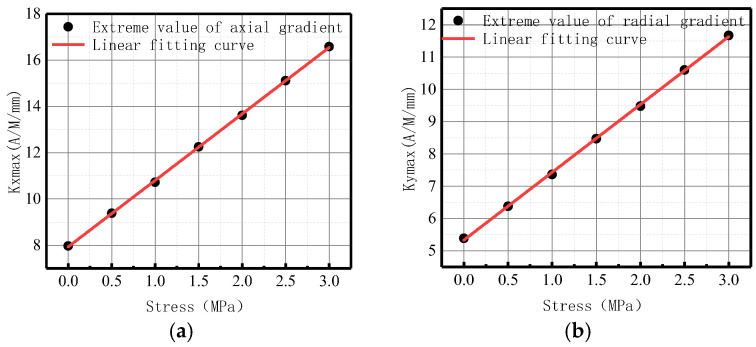
*Kmax* variation with internal pressure. (**a**) Axial component of *Kmax*; (**b**) radial component of *Kmax*.

The variation of *Kmax* with internal pressure for axial and radial components is illustrated in Figure 10.

It was revealed that in axial and radial components, *Kmax* linearly increased with the elevation of internal pressure. For an increase of pressure with an interval pressure of 0.5 MPa, the average change of *Kmax* for the axial component was 2.83 A/M/mm, and the average change of *Kmax* for the radial component was 1.95 A/M/mm.

#### 3.1.4. Gradient Energy Factor S(K) at Weld Varies

A new parameter, namely the gradient energy factor *S*(*K*)*,* is presented in this study. *S*(*K*) is the area surrounded by the gradient curve of magnetic field strength and the abscissa axis, and it can more directly reflect the degree of stress damage.

**Figure 11:**
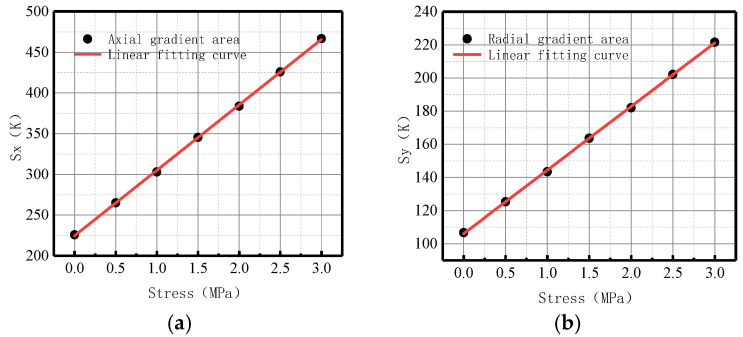
*S*(*K*) variation with internal pressure. (**a**) Axial component of *S*(*K*); (**b**) radial component of *S*(*K*).

The variations of the *S*(*K*) parameter with internal pressure in the axial and radial components are shown in Figure 11.

It was found that *S*(*K*) increased with the elevation of internal pressure. For an increase of pressure with an interval pressure of 0.5 MPa, the average change of *S*(*K*) for the axial component was 77.5, and the average change of *S*(*K*) for the radial component was 36.7.

The year-on-year growth rate is expressed as follows:

(20) $$\nu =\frac{\Delta A}{A_{1}}=\frac{A_{2}-A_{1}}{A_{1}}$$

where *ν* is the year-on-year growth rate, *A*_2_ denotes the secondary current value, *A*_1_ is the primary current value in the same period, and Δ*A* is the current increment.

Therefore, the year-on-year growth rate of energy factor *S*(*K*) for axial component *ν_x_* was 106.67%, and the growth rate of energy factor *S*(*K*) for radial component *ν_y_* was 109.43%. The radial growth rate *ν_y_* of *S*(*K*) was 3.24% higher than the axial growth rate *ν_x_*, indicating that the radial component of the magnetic signal at the weld of the ferromagnetic material was more sensitive to variations of stress.

It was revealed that the change of energy factor *S*(*K*) was larger and more obvious than that of *K* and *Kmax* in terms of order of magnitude and average change, which could more intuitively reflect the change of the magnetic signal. Therefore, *S*(*K*) can be used as a new parameter to comprehensively reflect the damage state of the weld.

### 3.2. Influences of Internal Pressure on the Weak Magnetic Signal of Weld Crack

The oil and gas pipelines must tolerate internal pressure during normal operation, and their combination with stress concentration may seriously affect the operational safety of the pipeline. Therefore, it is extremely necessary to analyze the influences of internal pressure on a pipeline’s weak magnetic signal.

**Figure 12:**
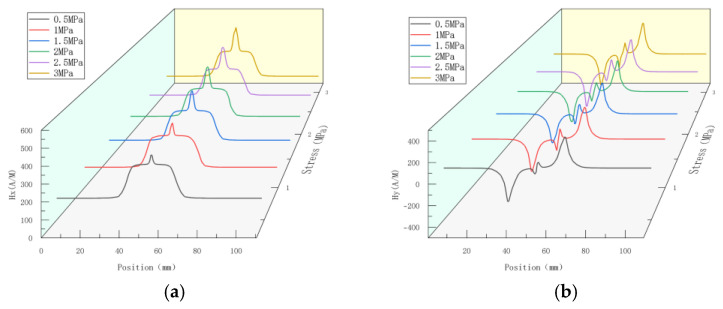
Magnetic signal of weld crack under different internal pressures. (**a**) Axial component of weak magnetic signal; (**b**) radial component of weak magnetic signal.

The initial residual stress obtained from the simulation in Section 2.3.2 was set as prestress, and an internal pressure of 0.5–3 MPa was applied to the pipe. The simulation was executed with a pressure interval of 0.5 MPa, and the influences of internal pressure on the weak magnetic field at the weld crack of the pipe were analyzed. The results of this test of the weak magnetic signal at the weld crack are shown in Figure 12:

According to the above-mentioned results of magnetic simulation, the peak values of the axial components and the peak-to-peak values of the radial components, gradient *K*, maximum gradient *Kmax*, and gradient energy factor *S*(*K*) of magnetic signal characteristic parameters were analyzed.

#### 3.2.1. Peak Values in Axial and Radial Components at Weld Crack

**Figure 13:**
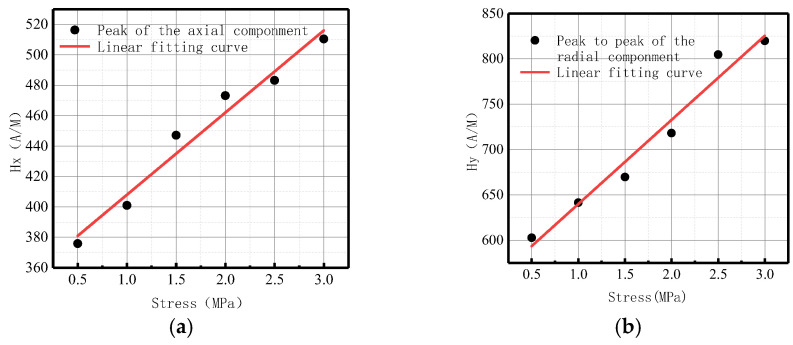
The peak values of weak magnetic signal at weld crack changes with internal pressures. (**a**) Peak values of the axial component; (**b**) peak-to-peak values of the radial component.

The variations of the peak values of the axial components and the peak-to-peak values of the radial components with internal pressure are shown in Figure 13.

It was revealed that the peak values in axial and radial components increased linearly with internal pressure. For an increase of pressure with an interval pressure of 0.5 MPa, the average change of peak value in the axial component was 83.33 A/M, and the average change of peak-to-peak value in the radial component was 137.5 A/M.

#### 3.2.2. Magnetic Field Strength Gradient K at Weld Crack

**Figure 14:**
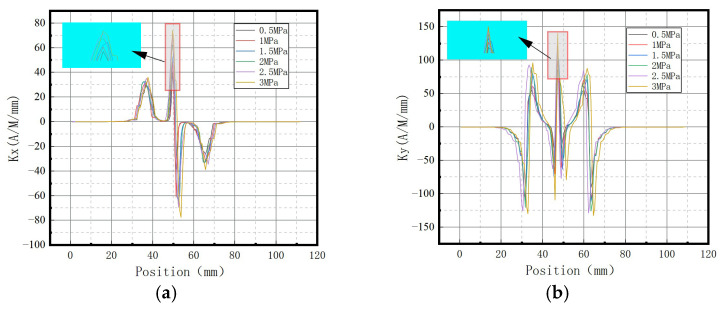
Magnetic signal gradient *K* at weld crack changes with internal pressures. (**a**) The axial gradient component *Kx*; (**b**) the radial gradient component *Ky*.

The variations of magnetic field strength gradient *K* for the axial and radial components under different internal pressures are shown in Figure 14.

It was found that the magnetic field strength gradient *K* for the axial and radial components gradually increased with elevation of internal pressure.

#### 3.2.3. Maximum Magnetic Field Strength Gradient Kmax at Weld Crack

**Figure 15:**
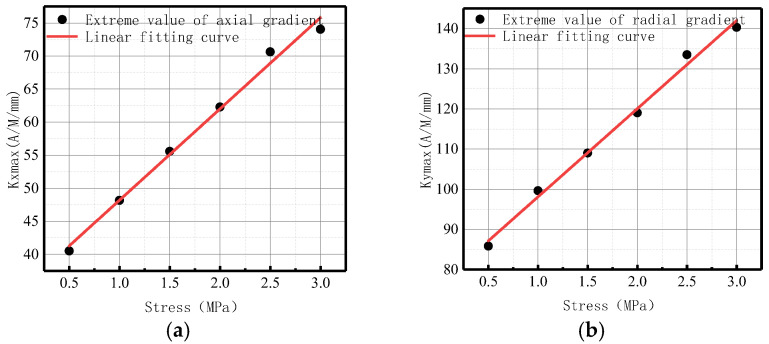
*Kmax* variations at weld crack with internal pressures. (**a**) Axial component of *Kmax*; (**b**) radial component of *Kmax*.

The changes of maximum magnetic field strength gradient *Kmax* for axial and radial components are illustrated in Figure 15.

It was revealed that in axial and radial components, *Kmax* linearly increased with the elevation of internal pressure. For an increase of pressure with an interval pressure of 0.5 MPa, the average change of *Kmax* for the axial component was 12.5 A/M/mm, and the average change of *Kmax* for the radial component was 23.3 A/M/mm.

#### 3.2.4. Gradient Energy Factor S(K) at Weld Crack

**Figure 16:**
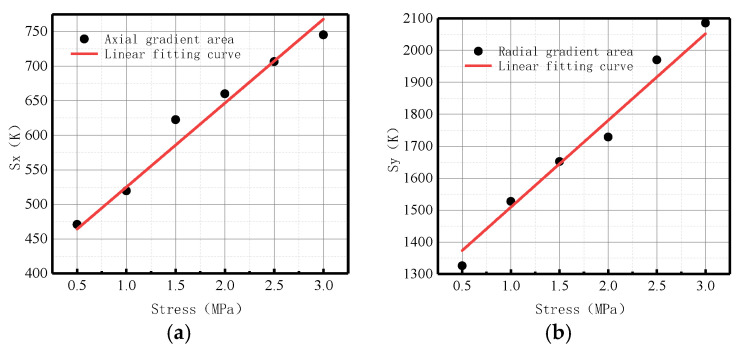
*S*(*K*) varies with the internal pressure. (**a**) Axial component of *S*(*K*); (**b**) radial component of *S*(*K*).

The variations of gradient energy factor *S*(*K*) in axial and radial components with internal pressure are shown in Figure 16.

It was found that the gradient energy factor *S*(*K*) in axial and radial components gradually increased with the elevation of internal pressure. For an increase of pressure with an interval pressure of 0.5 MPa, the average change of the gradient energy factor *S*(*K*) in the axial component was 125, and it was 340 in the radial component.

The year-on-year growth rate in the axial component *ν_x_* of energy factor *S*(*K*) was 59.57%; the year-on-year growth rate in the radial component *ν_y_* of energy factor *S*(*K*) was 61.54%. The year-on-year growth rate in the axial component was 1.97% higher than that in the radial component, indicating that the radial component of the magnetic signal at the weld crack of ferromagnetic materials was more sensitive to the variations of stress.

After Figure 16 was compared with Figure 14 and Figure 15, it was found that the gradient energy factor *S*(*K*), gradient *K*, and the maximum gradient *Kmax* followed the same pattern of variations. Moreover, the variation of *S*(*K*) was more significant than that of *K* and *Kmax*, indicating that *S*(*K*) can be replaced with the gradient factor to analyze the stress state at the weld.

### 3.3. Effects of Internal Pressure on Weak Magnetic Signal of Pipeline Base Metal Crack

**Figure 17:**
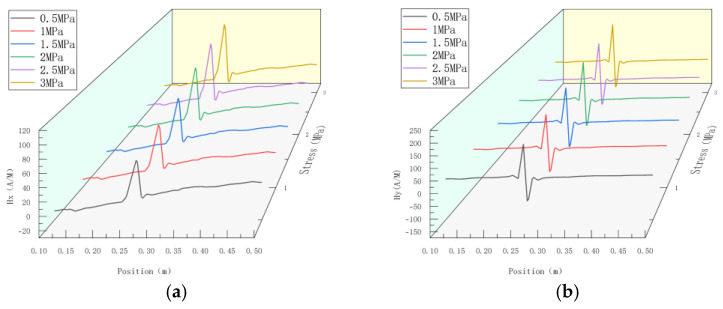
Magnetic signal of parent material crack under different internal pressure. (**a**) Axial component of weak magnetic signal; (**b**) radial component of weak magnetic signal.

A crack was created with the size of 2 mm × 0.95 mm × 1 mm (length × width × depth) on the pipe base metal (consistent with the weld base metal parameters). The results of the weak magnetic field simulation are shown in Figure 17.

According to the above-mentioned results of magnetic simulation, the peak values of the axial components and the peak-to-peak values of the radial components, gradient *K*, maximum gradient *Kmax*, and gradient energy factor *S*(*K*) of magnetic signal characteristic parameters were analyzed.

#### 3.3.1. Peak Values in Axial and Radial Components at the Crack of Base Metal

**Figure 18:**
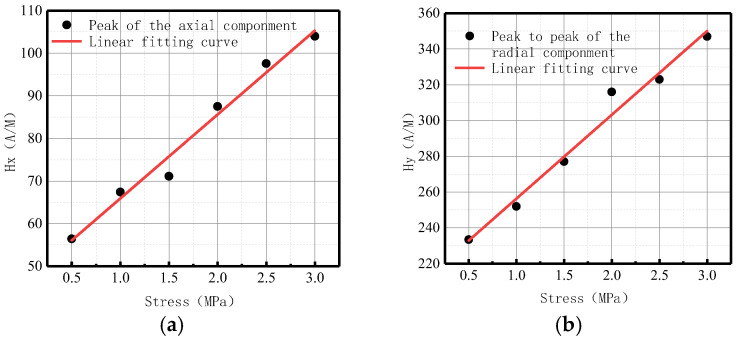
The peak values of weak magnetic signal at the crack of base metal changes with internal pressures. (**a**) Peak values of the axial component; (**b**) Peak-to-peak values of the radial component.

The variations of the peak values of the axial components and the peak-to-peak values of the radial components with internal pressure are shown in Figure 18.

It was revealed that peak values in axial and radial components increased linearly with the internal pressure. For an increase of pressure with an interval pressure of 0.5 MPa, the average change of peak value in the axial component was 17.67 A/M, and the average change of peak-to-peak value in the radial component was 52.17 A/M.

#### 3.3.2. Magnetic Field Strength Gradient K at the Crack of Base Metal

**Figure 19:**
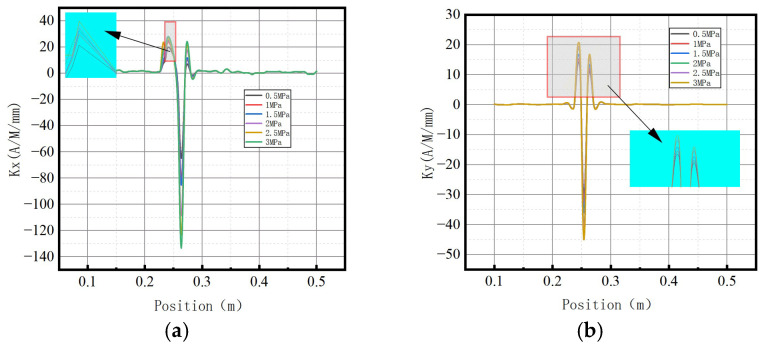
Magnetic signal gradient K at base metal crack changes with internal pressures. (**a**) The axial gradient component *Kx*; (**b**) the radial gradient component *Ky*.

The variations of magnetic field strength gradient *K* for the axial and radial components under different internal pressures are shown in Figure 19.

It was found that magnetic field strength gradient *K* for the axial and radial components gradually increased with the elevation of internal pressure.

#### 3.3.3. Maximum Magnetic Field Strength Gradient Kmax at the Crack of Base Metal

**Figure 20:**
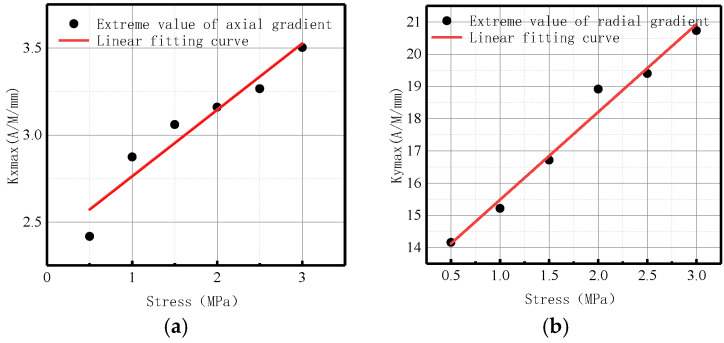
*Kmax* variations at base metal crack with internal pressures. (**a**) Axial component of *Kmax*; (**b**) radial component of *Kmax*.

The changes of maximum magnetic field strength gradient *Kmax* for axial and radial components are illustrated in Figure 20.

It was revealed that in axial and radial components, *Kmax* linearly increased with the elevation of internal pressure. For an increase of pressure with an interval pressure of 0.5 MPa, the average change of *Kmax* for axial component was 0.58 A/M/mm, and the average change of *Kmax* for radial component was 3.5 A/M/mm.

#### 3.3.4. Gradient Energy Factor S(K) at the Crack of Base Metal

**Figure 21:**
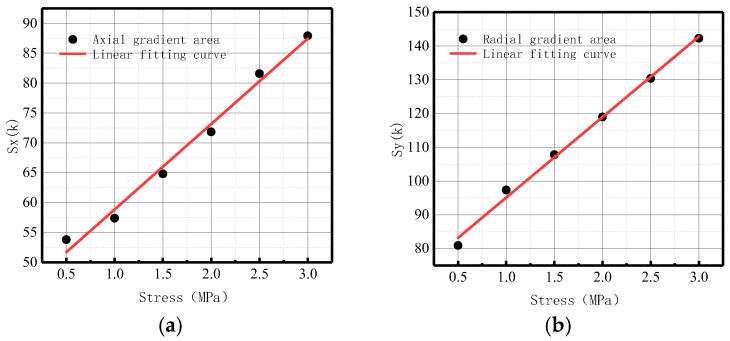
*S*(*K*) varies with the internal pressure. (**a**) Axial component of *S*(*k*); (**b**) radial component of *S*(*k*).

The variations of gradient energy factor *S*(*K*) in axial and radial components with internal pressure are shown in Figure 21.

It was revealed that *S*(*K*) in both axial and radial components increased linearly with the elevation of internal pressure. For an increase of pressure with an interval pressure of 0.5 MPa, the average change of *S*(*K*) in the axial component was 14.5, and it was 23.67 in the radial component. The year-on-year growth rate in the axial component *ν_x_* of energy factor *S*(*K*) was 64.15%; the year-on-year growth rate in the radial component *ν_y_* of energy factor *S*(*K*) was 77.5%. The year-on-year growth rate in the axial component was 13.35% higher than that in the radial component, indicating that the radial component of the magnetic signal at the weld crack of ferromagnetic materials was more sensitive to the variations of stress. From the perspective of magnitude and average change, *S*(*K*) had a greater variation than *K* and *Kmax*, and the variation may be more obvious.

According to the above-mentioned parametric analysis of the weld, weld cracks, and base metal cracks, it was noted that the magnitude and average change of the proposed new parameter *S*(*K*) were larger than those of *K* and *Kmax*, thus, *S*(*K*) can be used as a new parameter to comprehensively assess the stress-induced damage of pipelines.

### 3.4. Comprehensive Analysis of Characteristic Parameters of Weak Magnetic Signals

**Figure 22:**
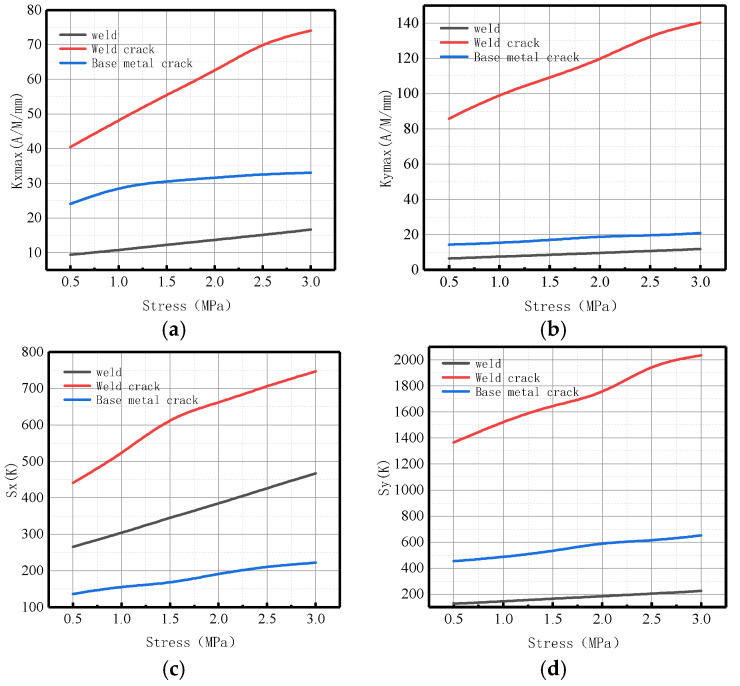
Variations of *Kmax* and *S*(*K*) parameters calculated by simulation under three conditions. (**a**) Axial component of *Kmax*; (**b**) radial component of *Kmax*. (**c**) Axial component of *S*(*k*); (**d**) radial component of *S*(*k*).

The variations of *Kmax* and *S*(*K*) parameters in the weld, weld crack, pipe base metal crack, and pipe damage were comprehensively analyzed (Figure 22).

It can be observed from the above-illustrated figure that during weld crack detection, the two parameters had generally great changes, and the order of magnitude of *S*(*K*) was greater than that of *Kmax*, accompanied by more significant changes.

**Figure 23:**
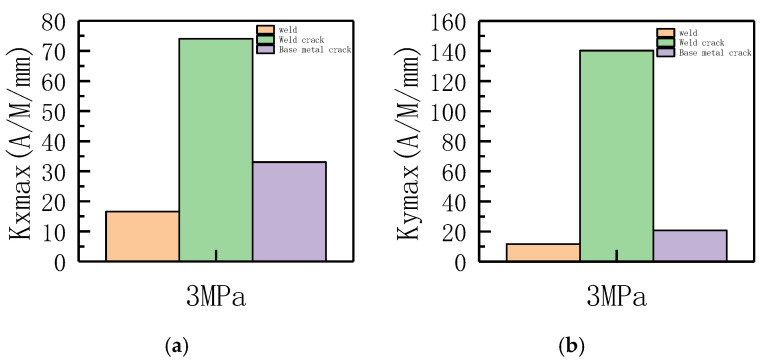

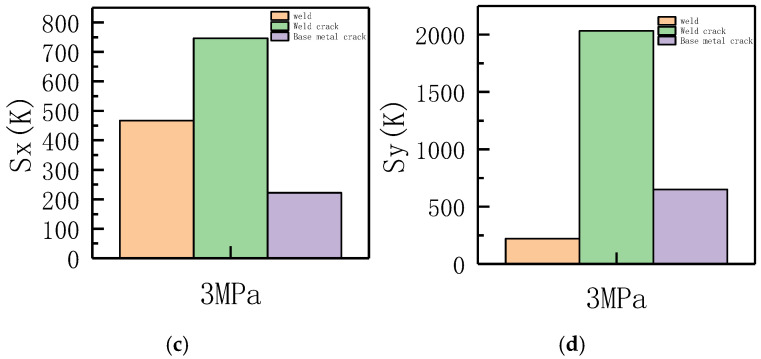
Variations of *Kmax* and S(K) parameters at 3 MPa. (**a**) Axial component of *Kmax*; (**b**) radial component of *Kmax*. (**c**) Axial component of *S*(*k*); (**d**) radial component of *S*(*k*).

The variations of *Kmax* and *S*(*K*) parameters under the same internal pressure of 3 MPa are shown in Figure 23.

It can be observed from the above-illustrated figure that during weld crack detection, *Kmax* and *S*(*K*) significantly changed, the stress concentration was large, and the risk of failure was high. Therefore, the pipe weld crack should be detected in time with this method.

## 4. Experiment and Analysis

In order to study the weak magnetic signal characteristics of weld cracks in long oil and gas pipelines and verify the reliability of the numerical model, a pipeline pressurization experiment was designed to analyze the changes of the weak magnetic signal characteristic parameters of pipeline welds, weld cracks, and base metal cracks. The experimental results verified the reliability of the numerical model.

### 4.1. Experimental Materials

The experimental material was a section of X70 welded pipe with artificial cracks. The overall length of the pipe was 6000 mm, the diameter was 1012 mm, and the wall thickness was 14.5 mm.

### 4.2. Experimental Equipment

**Figure 24:**
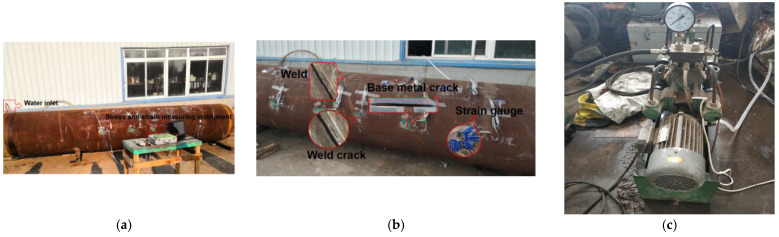
Experimental setup. (**a**) Experimental arrangement; (**b**) weld, weld crack, and base metal crack; (**c**) pressure suppression equipment.

The test equipment was TSC-2M-8 made by the Russian Power Diagnosis Company (Moscow, Russia). The lift off value was set to 1 mm. The weak magnetic signal under different internal pressures was measured perpendicular to the weld and crack. A diagram of the test measurement is shown in Figure 24.

### 4.3. Experimental Methods

First, both ends of the pipe and weld were blocked with a water nozzle with a diameter of φ 50 mm. One end was considered the water inlet, and the other end was the water outlet. During the experiment, the pressure pump was used to inject water into the water inlet to simulate the working state of the pipeline during normal operation, and the water pressure sensor was installed to monitor the change of water pressure in the pipeline to prevent cracking in pipelines. A strain gauge was attached to the crack tip, and the stress at the crack tip was detected with the stress and strain measurement devices. Once the strain at the crack tip exceeded the preset threshold, the strain gauge would trigger an alarm and terminate water injection into the pipes to reduce the internal pressure of the pipes and ensure safety.

When the pipeline was pressurized, the pressure was held for 30 min with pressure increments of 0.5 MPa. When stress distribution was stable, the weak magnetic signals of the weld, weld crack, and base metal crack were measured.

### 4.4. Analysis of the Experimental Results

#### 4.4.1. Weak Magnetic Signal of Weld

**Figure 25:**
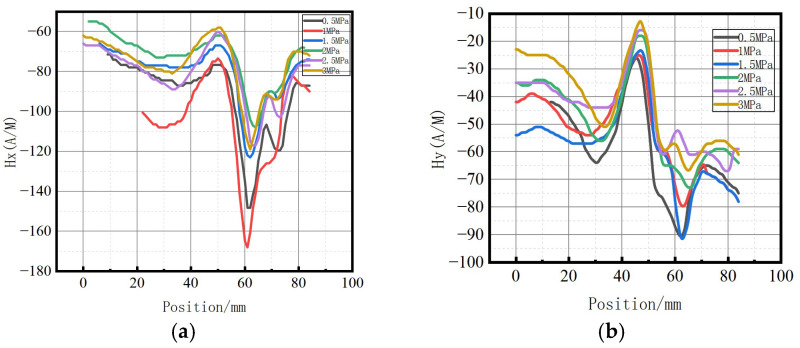
Magnetic signal changes at the weld with internal pressures. (**a**) Axial component of weak magnetic signal; (**b**) radial component of weak magnetic signal.

The variation pattern of weak magnetic signal characteristics at the pipe weld is shown in Figure 25.

According to the experimental results, the magnetic signal characteristic parameters, such as axial peak value and radial peak-to-peak value, gradient *K*, maximum gradient *Kmax*, and gradient energy factor *S*(*K*), were analyzed.

1. Peak values in axial and radial components

**Figure 26:**
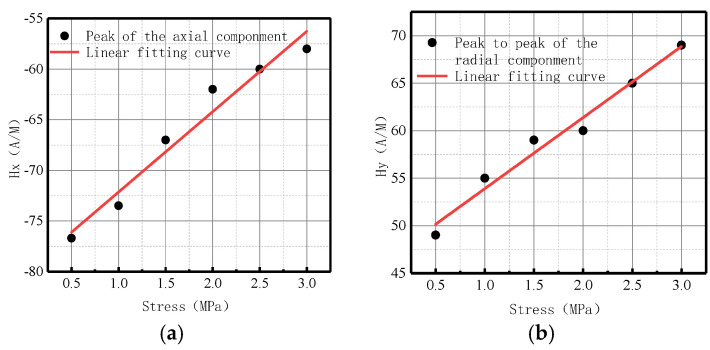
Peak value variations with internal pressure. (**a**) Peak values of the axial component; (**b**) peak-to-peak values of the radial component.

The fitting results of axial peak value and radial peak-to-peak value versus internal pressure are illustrated in Figure 26.

It can be observed from the above-illustrated figure that the peak value of the axial component and the peak-to-peak value of the radial component increased linearly with the elevation of internal pressure.

2. Magnetic field strength gradient *K*

**Figure 27:**
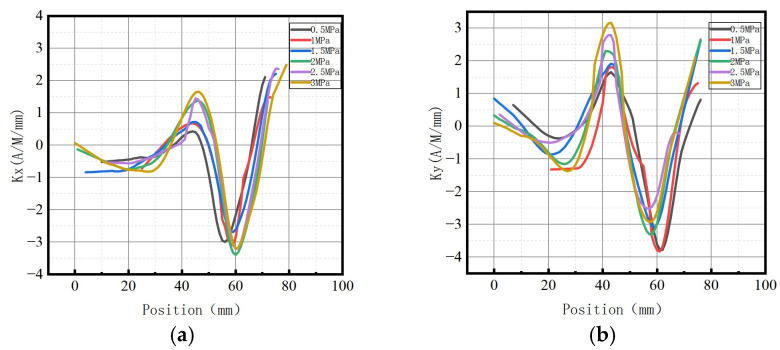
Variations of the magnetic field strength gradient *K* at the weld under different internal pressures. (**a**) The axial gradient component *Kx*; (**b**) the radial gradient component *Ky*.

The variations of the magnetic field strength gradient *K* under different internal pressures are shown in Figure 27.

It can be observed that the magnetic field intensity gradient *K* increased with the elevation of internal pressure, which is consistent with the numerical data.

3. Maximum magnetic field intensity gradient *Kmax*

**Figure 28:**
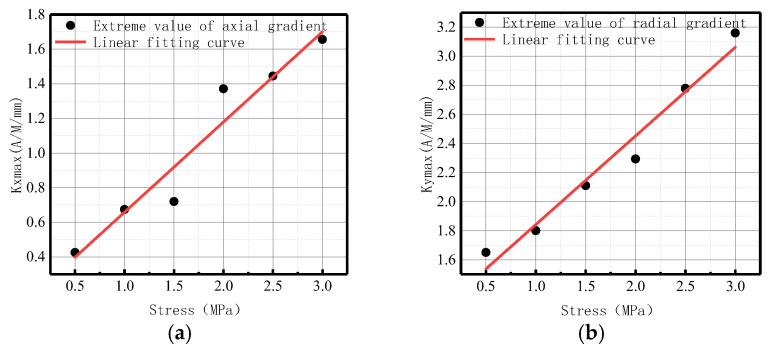
Variations of *Kmax* at the weld under different internal pressures. (**a**) Axial component of *Kmax*; (**b**) radial component of *Kmax*.

The variations of *Kmax* under different internal pressures are shown in Figure 28.

It can be observed that with the increase of internal pressure, *Kmax* became larger, which is consistent with the numerical data.

4. Gradient energy factor *S*(*K*)

**Figure 29:**
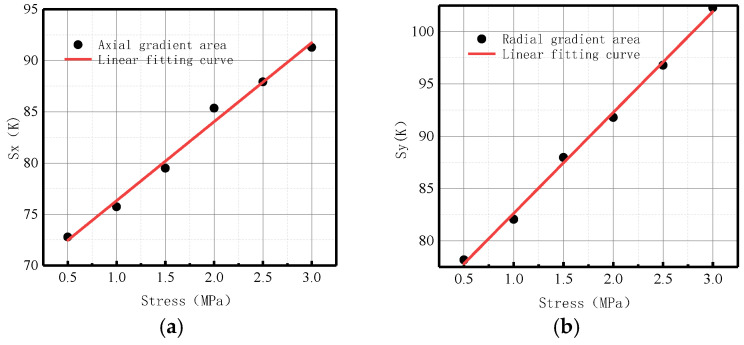
Variations of *S*(*K*) at the weld under different internal pressures. (**a**) Axial component of *S*(*k*); (**b**) radial component of *S*(*k*).

Figure 29 shows the variations of *S*(*K*) enclosed by the magnetic field intensity gradient curve and the abscissa axis under different internal pressures.

It can be observed from the above-illustrated fitting curve that *S*(*K*) increased with the elevation of internal pressure, which is consistent with the numerical data. The year-on-year growth rate of axial component *ν_x_* of energy factor *S*(*K*) was 26.39%; the year-on-year growth rate of radial component *ν_y_* of energy factor *S*(*K*) was 30.77%. The year-on-year growth rate of the radial component was larger than that of the axial component, and the radial component of the magnetic signal at the weld crack was more sensitive to the variations of stress, thus verifying the correctness of the numerical data.

In terms of order of magnitude, *S*(*K*) was greater than *K* and *Kmax*, and the degree of variation was more obvious. The experimental results were consistent with the numerical data, and it was confirmed that the new parameter *S*(*K*) can more intuitively and comprehensively indicate weld crack-induced failures in pipelines.

#### 4.4.2. Weld Crack

**Figure 30:**
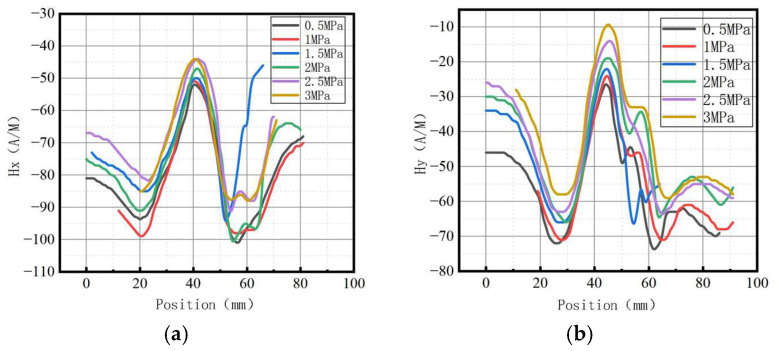
Magnetic signal changes at weld crack with internal pressures. (**a**) Axial component of weak magnetic signal; (**b**) radial component of weak magnetic signal.

The variation pattern of weak magnetic signal characteristics at the pipe weld crack is shown in Figure 30.

According to the experimental results, the magnetic signal characteristic parameters, such as axial peak value and radial peak-to-peak value, gradient *K*, maximum gradient *Kmax*, and gradient energy factor *S*(*K*) were analyzed.

1. Peak values in axial and radial components

**Figure 31:**
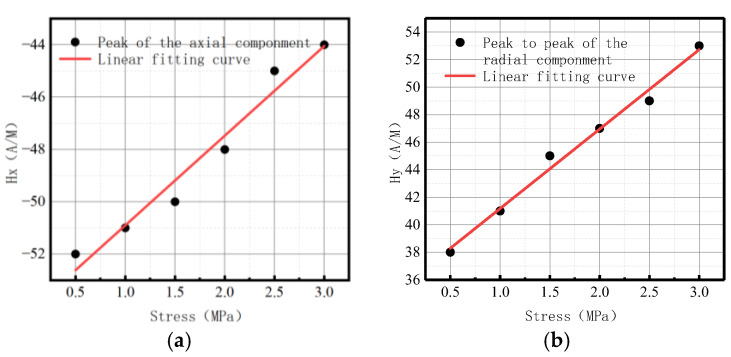
Peak value varies at weld crack with internal pressures. (**a**) Peak values of the axial component; (**b**) peak-to-peak values of the radial component.

The fitting results of axial peak value and radial peak-to-peak value versus internal pressure are illustrated in Figure 31.

It can be observed from the above-illustrated figure that the peak value of the axial component and the peak value of the radial component increased linearly with the elevation of internal pressure.

2. Magnetic field strength gradient *K*

**Figure 32:**
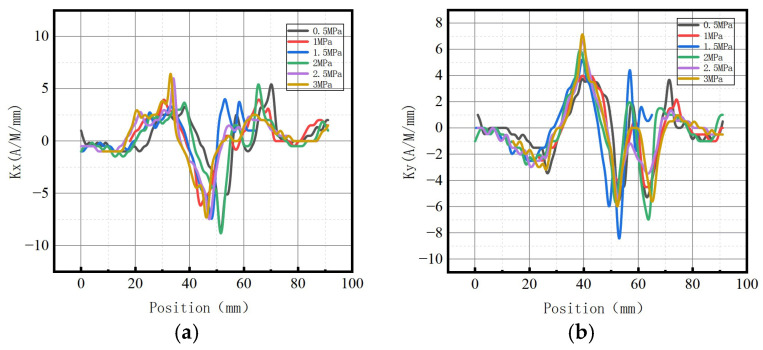
Variations of the magnetic field strength gradient *K* at weld crack under different internal pressures. (**a**) The axial gradient component *Kx*; (**b**) the radial gradient component *Ky*.

The variations of the magnetic field strength gradient *K* under different internal pressures are shown in Figure 32.

It can be observed that the magnetic field intensity gradient *K* increased with the elevation of internal pressure, which is consistent with numerical data.

3. Maximum magnetic field intensity gradient *Kmax*

**Figure 33:**
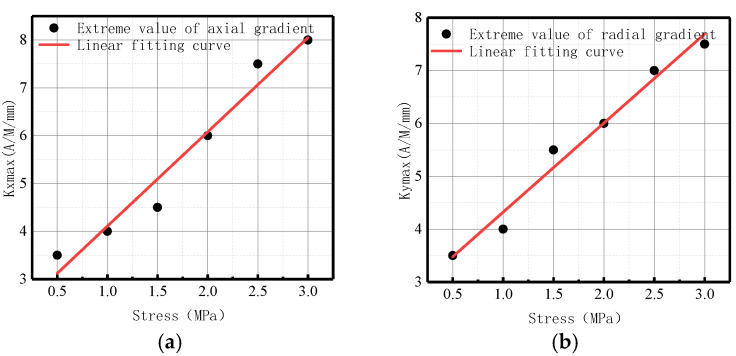
Variations of *Kmax* at weld crack under different internal pressures. (**a**) Axial component of *Kmax*; (**b**) radial component of *Kmax*.

The variations of *Kmax* under different internal pressures are shown in Figure 33.

It can be observed that with the increase of internal pressure, *Kmax* became larger, which is consistent with the numerical data.

4. Gradient energy factor *S*(*K*)

**Figure 34:**
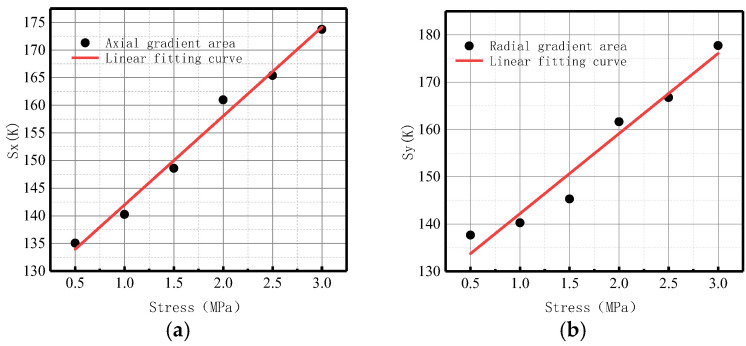
Variations of *S*(*K*) at weld crack under different internal pressures. (**a**) Axial component of *S*(*k*); (**b**) radial component of *S*(*k*).

Figure 34 shows the variations of the *S*(*K*) enclosed by the magnetic field intensity gradient curve and the abscissa axis under different internal pressures.

It was revealed that *S*(*K*) increased with the elevation of internal pressure, which is consistent with the numerical data. The year-on-year growth rate of axial component *ν_x_* of *S*(*K*) was 29.63%; the year-on-year growth rate of radial component *ν_y_* of *S*(*K*) was 30.88%. The year-on-year growth rate of the radial component was higher than that of the axial component, and the radial component of the magnetic signal at the weld crack was more sensitive to the variations of stress, thus verifying the correctness of the numerical data.

#### 4.4.3. Crack of Pipe Base Metal

**Figure 35:**
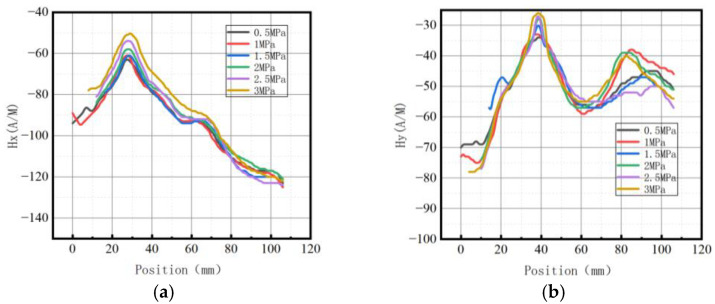
Magnetic signal changes at the crack of base metal with internal pressures. (**a**) Axial component of weak magnetic signal; (**b**) radial component of weak magnetic signal.

The variation pattern of weak magnetic signal characteristics at the crack in the pipe base metal is illustrated in Figure 35.

According to the experimental results, the magnetic signal characteristic parameters, such as axial peak value and radial peak-to-peak value, gradient *K*, maximum gradient *Kmax,* and gradient energy factor *S*(*K*) were analyzed.

1. Peak values in axial and radial components

**Figure 36:**
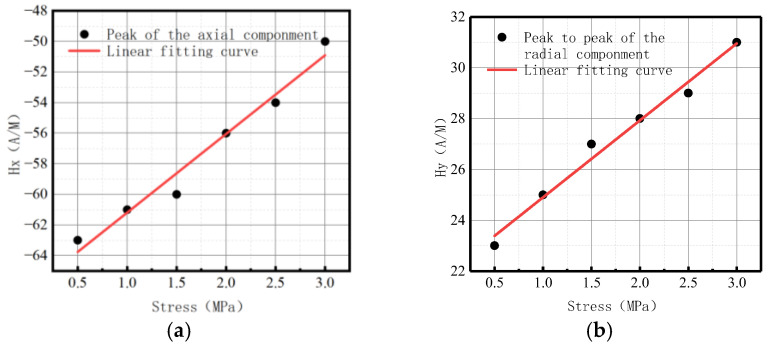
Peak value varies at the crack of base metal with internal pressures. (**a**) Peak values of the axial component; (**b**) peak-to-peak values of the radial component.

The fitting results of axial peak value and radial peak-to-peak value versus internal pressure are illustrated in Figure 36.

It can be observed from the above-illustrated figure that the peak value of the axial component and the peak-to-peak value of the radial component increased linearly with the elevation of internal pressure.

2. Magnetic field strength gradient *K*

**Figure 37:**
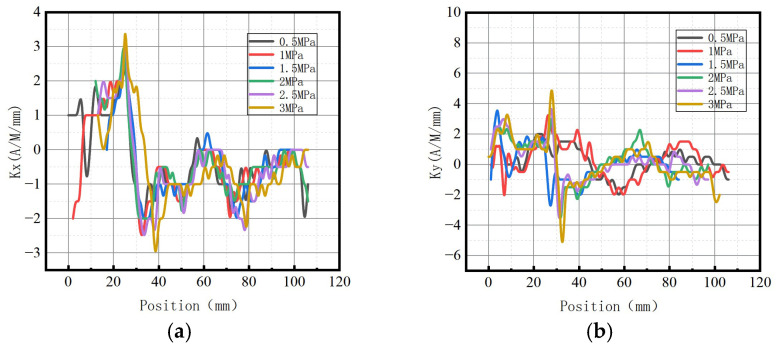
Variations of the magnetic field strength gradient *K* at the crack of base metal under different internal pressures. (**a**) The axial gradient component *Kx*; (**b**) the radial gradient component *Ky*.

The variations of the magnetic field strength gradient *K* under different internal pressures are shown in Figure 37.

It can be observed that the magnetic field intensity gradient *K* increased with the elevation of internal pressure, which is consistent with numerical data.

3. Maximum magnetic field intensity gradient *Kmax*

**Figure 38:**
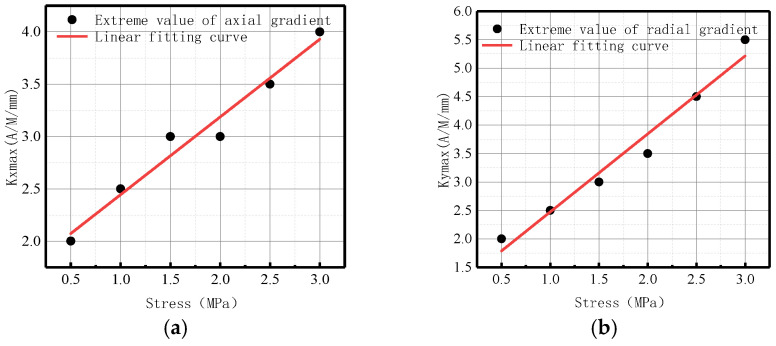
Variations of *Kmax* at the crack of base metal under different internal pressures. (**a**) Axial component of *Kmax*; (**b**) radial component of *Kmax*.

The variations of *Kmax* under different internal pressures are shown in Figure 38.

It can be observed that with the increase of internal pressure, *Kmax* became larger, which is consistent with the numerical data.

4. Gradient energy factor *S*(*K*)

**Figure 39:**
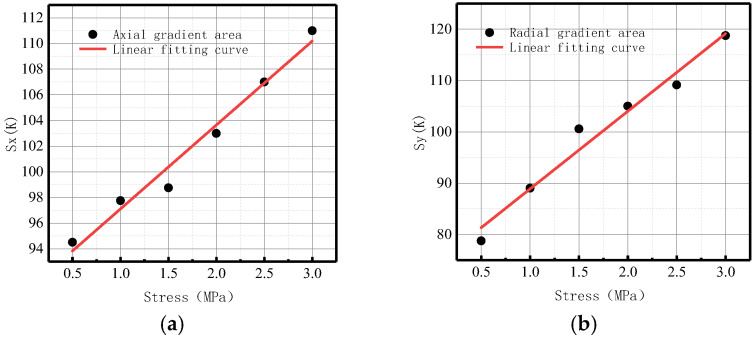
Variations of *S*(*K*) at the crack of base metal under different internal pressures. (**a**) Axial component of *S*(*k*); (**b**) radial component of *S*(*k*).

Figure 39 shows the variations of the *S*(*K*) enclosed by the magnetic field intensity gradient curve and the abscissa axis under different internal pressures.

It was revealed that *S*(*K*) increased with the elevation of internal pressure, which is consistent with the numerical data. From the perspective of magnitude, *S*(*K*) was greater than *K* and *Kmax*, and the degree of variation was more obvious. The experimental results were consistent with the numerical data. The year-on-year growth rate of axial component *ν_x_* of energy factor *S*(*K*) was 18.09%; the year-on-year growth rate of radial component *ν_y_* of energy factor *S*(*K*) was 50%. The year-on-year growth rate of the radial component was larger than that of the axial component, and the radial component of the magnetic signal at the base metal crack was more sensitive to the variations of stress, thus verifying the correctness of the numerical data.

According to the parametric analysis of the weld, weld crack, and base metal crack, it was noted that the order of magnitude and average change of the new parameter *S*(*K*) were larger than *K* and *Kmax*, thus, *S*(*K*) can be used as a new parameter to comprehensively indicate weld crack-induced failures in pipelines.

### 4.5. Comprehensive Analysis of Characteristic Parameters

**Figure 40:**
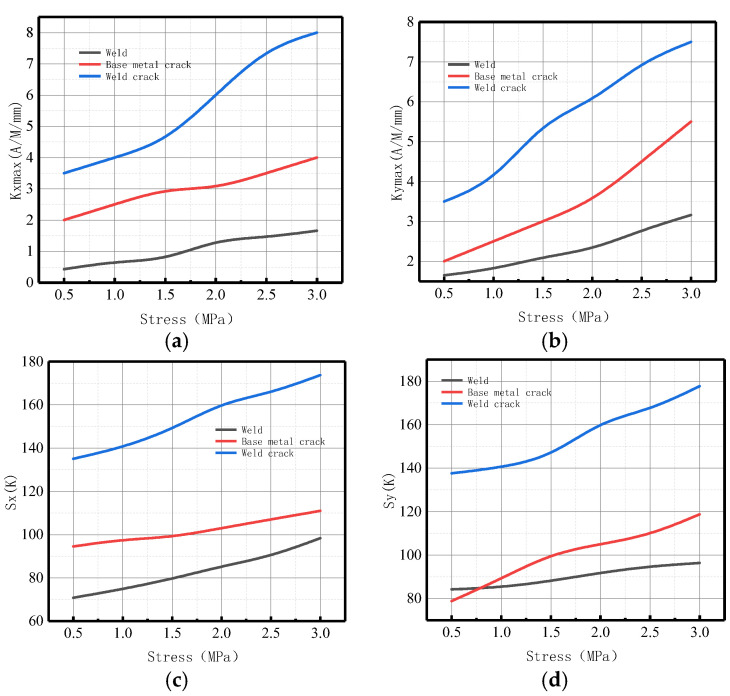
Variations of *Kmax* and *S*(*K*) parameters calculated by experiment under three conditions. (**a**) Axial component of *Kmax*; (**b**) radial component of *Kmax*. (**c**) Axial component of *S*(*k*); (**d**) radial component of *S*(*k*).

The changes of *Kmax* and energy factor *S*(*K*) as related to the parameters of the weld, weld crack, and base metal crack were comprehensively analyzed, as displayed in Figure 40.

It can be observed from the above-illustrated figure that during weld crack detection, the variations of the two parameters were large on the whole, and the order of magnitude and degree of variation of *S*(*K*) were greater than those of *Kmax*. Therefore, the energy factor *S*(*K*) can be used as a new parameter to comprehensively indicate weld crack-induced failure in a pipeline, verifying the correctness of the numerical data.

**Figure 41:**
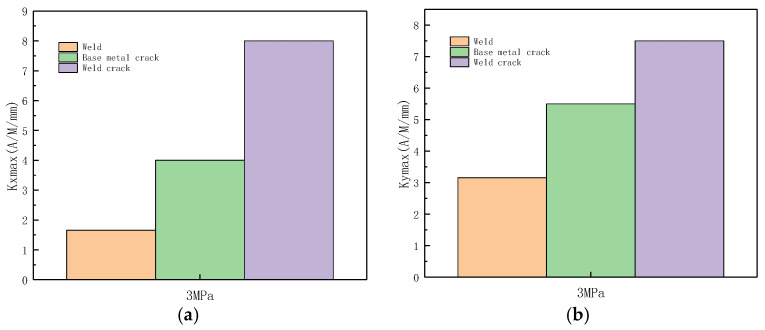

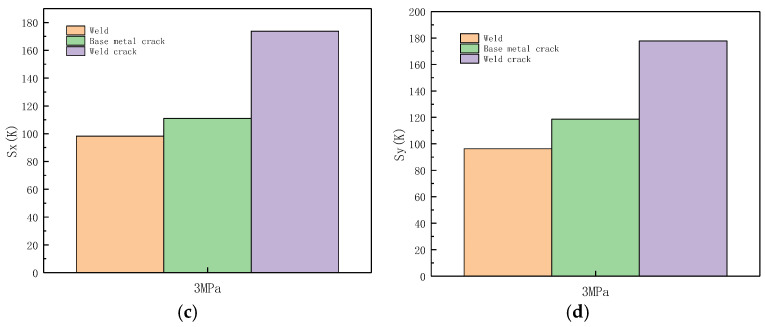
Variations of *Kmax* and *S*(*K*) parameters at an internal pressure of 3 MPa. (**a**) Axial component of *Kmax*; (**b**) radial component of *Kmax*. (**c**) Axial component of *S*(*k*); (**d**) radial component of *S*(*k*).

The variations of *Kmax* and *S*(*K*) parameters under the same internal pressure of 3 MPa are shown in Figure 41.

It can be observed from the above-illustrated figure that during weld crack detection, the *Kmax* and *S*(*K*) parameters significantly varied, indicating that the stress concentration was noticeable; thus, the weld crack was the most dangerous and should be detected in time.

## 5. Conclusions

Through numerical simulation and experimental verification, the following conclusions could be drawn.

When the pipeline is welded, the microstructure of the material at the weld changes, thus resulting in a large amount of martensite. The linear relationship between the ferromagnetism of the material and the content of martensite is the cause of the magnetic signal at the weld.

Under the action of internal pressure, the magnetic signal parameters (*K*, *Kmax*, and energy factor *S*(*K*)) of the weld, weld crack, and base metal crack increased with the elevation of internal pressure. The average variation of the newly proposed parameter, energy factor *S*(*K*), was larger, which could more intuitively indicate weld crack-induced failure in a pipeline.

Under internal pressure, the radial year-on-year growth rate *ν_y_* of the energy factor *S*(*K*) of the weld, weld crack and base metal crack were greater than the axial year-on-year growth rate *ν_x_* (*ν_y_* at the weld was 3.24% higher than *ν_x_*, *ν_y_* at the weld crack was 1.97% higher than *ν_x_*, and *ν_y_* at the base metal crack was 13.35% higher than *ν_x_* in this study), indicating that the radial component of the magnetic signal is more sensitive to variations of stress.

Subsequently, more in-depth research will be carried out in the field of micro-cracks in pipeline welds to realize real-time warnings of micro-cracks and avoid accidents.

## Data Availability

Not applicable.
